# Stability data of FlgD from *Helicobacter pylori* and structural comparison with other homologs

**DOI:** 10.1016/j.dib.2016.02.068

**Published:** 2016-03-04

**Authors:** Ivana Pulić, Laura Cendron, Marco Salamina, Patrizia Polverino de Laureto, Dubravka Matković-Čalogović, Giuseppe Zanotti

**Affiliations:** a University of Zagreb, Faculty of Science, Department of Chemistry, Division of General and Inorganic Chemistry, Horvatovac 102a, Zagreb 10000, Croatia; b Department of Biomedical Sciences, University of Padua, Via Ugo Bassi 58/B, Padua 35131, Italy; c Department of Biology, University of Padua, Via Ugo Bassi 58/B, Padua 35131, Italy; d Department of Pharmaceutical Sciences, University of Padua, Via Marzolo 5, Padua 35131, Italy

## Abstract

Flagellin component D (FlgD) from *Helicobacter pylori* is involved in the assembly of the hook of flagella, helical tubular structures that provide motility in non-filamentous bacteria. Data provided in this article refer to *Hp*FlgD from strains 26695 (*Hp*FlgD_26695) and G27 (*Hp*FlgD_G27). Within this article, information on the secondary structure content and different type of interfaces found in the two crystal forms of *Hp*FlgD (monoclinic, *Hp*FlgD_m and tetragonal, *Hp*FlgD_t) are provided, as well as the list of the hydrogen bonds between monomers that are relevant for their assembly into a tetramer. Additionally, data involving investigation of the size of *Hp*FlgD in the solution and the crystallized *Hp*FlgD are presented, “Crystal structure of truncated FlgD from the human pathogen *Helicobacter pylori*” [1]. The superposition of the different domains of *Hp*FlgD (Fn-III and tudor domains) with the similar domains found in other species is shown, as well as the superposition of *Hp*FlgD and modeled *Hp*FlgE (flagellar hook protein).

**Specifications Table**

**Table:** 

Subject area	*Chemistry*
More specific subject area	*Protein crystallography and biophysics*
Type of data	*Table, text file, graph, figure*
How data was acquired	*Mass spectroscopy (*quadrupole-TOF spectrometer, RP-HPLC), X-ray diffraction (Swiss Light Source, SLS)
Data format	*Raw, analyzed*
Experimental factors	Crystals of native *Hp*FlgD_26695 were dissolved in the appropriate buffer, as well as a sample of *Hp*FlgD_26695 protein solution, and were run on a SDS-PAGE. The isolated bands were in gel digested with trypsin and the extracted peptides were further analyzed with nano-electrospray ionization mass spectrometry (nano-ESI MS).
Experimental features	The full length *Hp*FlgD_G27 monomer mass was determined by reverse phase chromatography (RP-HPLC). Mass measurements were performed with a quadrupole-TOF spectrometer and the obtained spectra was further analyzed using the MASSLYNX software.
Data source location	*Padua, Italy and – for mass spectroscopy dataVilligen, Switzerland, SLS − for crystallography data*
Data accessibility	*Data is with this article.*

**Value of the data**

• Providing the data on the protein stability can benefit other researchers willing to follow the same techniques.
• Interpretation of differences and similarities in the structural organization of FlgD homologs can be useful for future investigations on the role of FlgD in flagellar biogenesis.
• Previously unreported data on the secondary structure composition of the full length FlgD.

## 1 Data

This article presents data on the *Hp*FlgD stability in terms of the protein size. This investigation was done in order to understand which part of the degraded protein crystallized. The data is based on the CD and mass spectra analysis (RP-HPLC, nano-ESI). In addition, comparison of different types of interfaces found in the crystal structures of the two crystal forms of *Hp*FlgD [1] are given, as well as the amino acid residues responsible for the quaternary structure assembly. The difference between the domain orientation in *Hp*FlgD and the similar domains in other organisms is also shown.

## 2 Experimental design, materials and methods

**Fig. 1:**
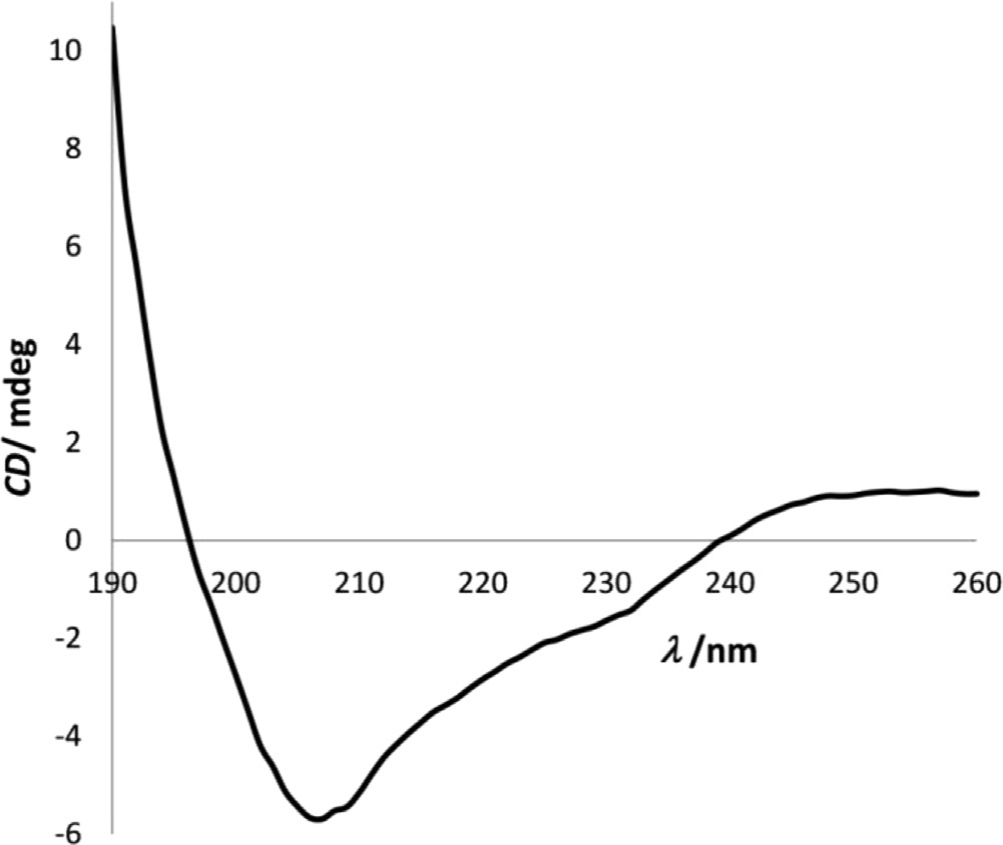
CD spectrum of the full length *Hp*FlgD_G27 in the far UV region (190–260 nm) presented as a CD signal in milidegrees.

Secondary structure analysis of diluted *Hp*FlgD (2 mg mL^−1^) was performed by circular dichroism (CD) using a spectropolarimeter (Jasco Analytical Instruments) in the far UV region (190–260 nm), Fig. 1. Afterwards, the data were deconvoluted using software CDNN [2] and are shown as contributions of the various components to the protein secondary structure (Table 1).

**Table 1:** CD data of the full length *Hp*FlgD_G27 analysed by the secondary structure analysis software, CDNN. Deconvoluted results are shown as contributions of the various components to the protein secondary structure.

**Secondary structure element**	**%**
Helix	12.8
Antiparallel β sheet	25.2
Parallel β sheet	5.0
β turn	22.8
Random coil	24.8

**Fig. 2:**
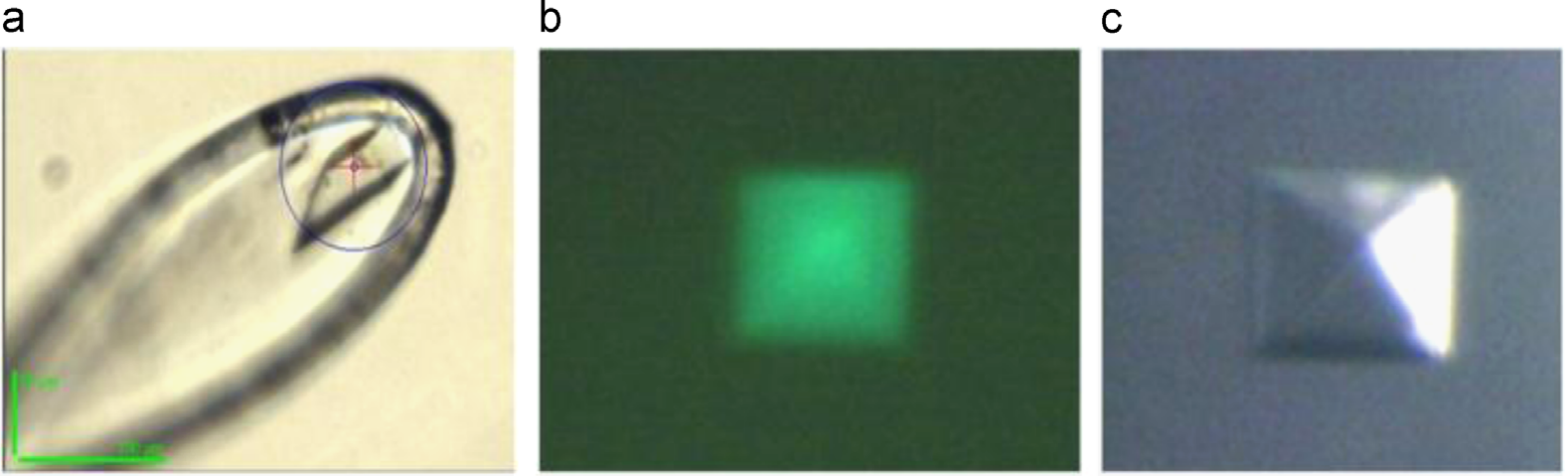
(a) Monoclinic crystal of native *Hp*FlgD_G27 and (b, c) tetragonal crystal of native *Hp*FlgD_26695. Picture (b) was captured under the microscope using a fluorescence excitation filter (CWL/BW=450/50 nm).

**Fig. 3:**
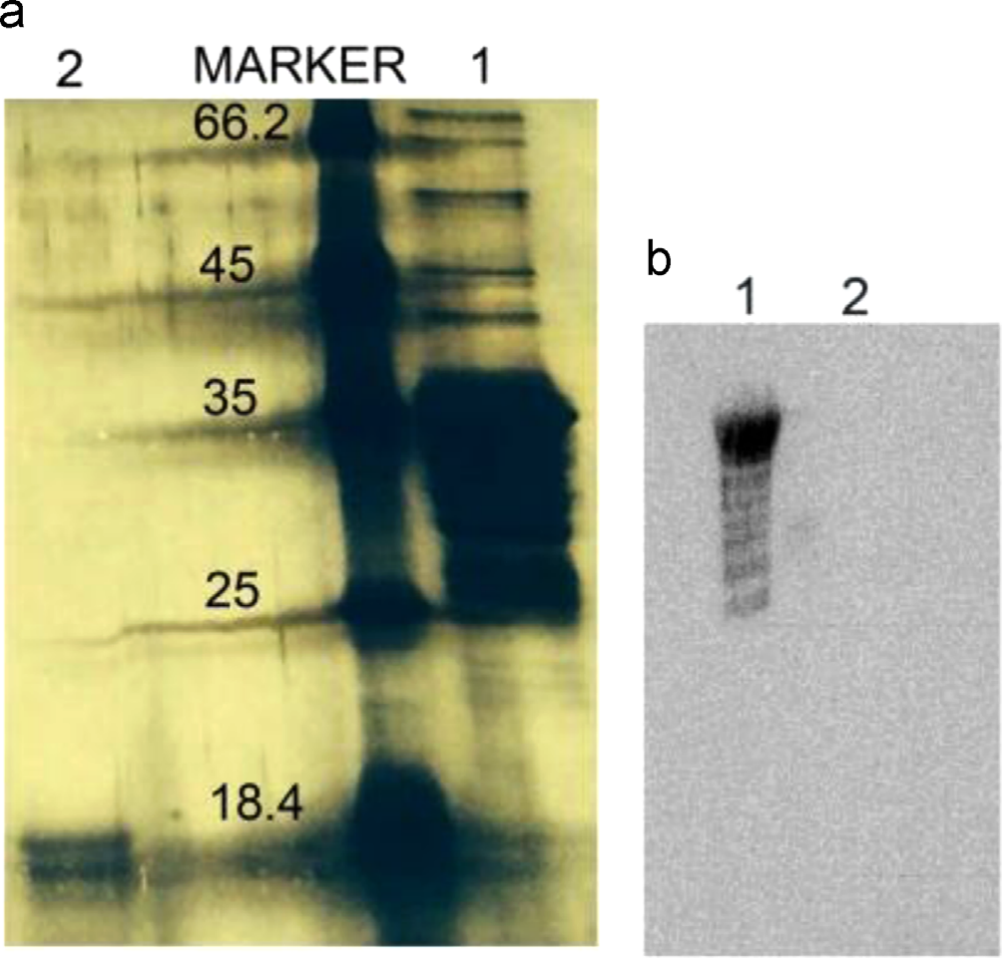
(a) SDS-PAGE; (b) Western blot against His tag at the C-terminal end. Lane 1 − full length *Hp*FlgD_26695 (top band) with initial degradation products (lower bands), Lane 2 – dissolved crystal of *Hp*FlgD_t.

**Fig. 4:**
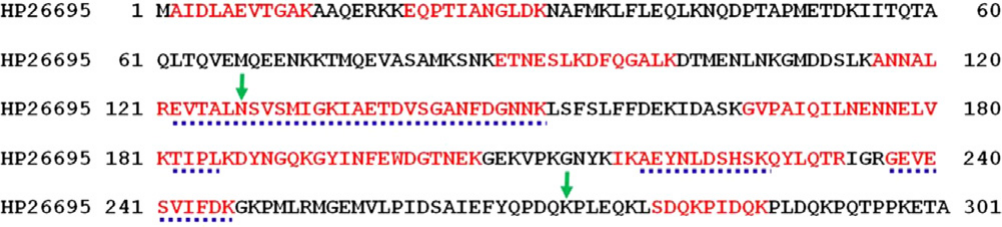
Results of the mass spectrometry: the peptides found in the full length *Hp*FlgD_26695 are bolded in red, while the peptides found in the tetragonal crystal of *Hp*FlgD_26695 are indicated by blue dotted lines. The starting and ending residues found in the crystal structure of *Hp*FlgD_t are marked with green arrows.

The level of degradation of *Hp*FlgD_26695 and crystallized *Hp*FlgD_26695 was monitored by the SDS-PAGE. The sample from the crystal of the tetragonal form of *Hp*FlgD_26695 (Fig. 2b and c) was prepared by dissolving the crystal in the SDS-PAGE loading buffer. This sample together with a full length *Hp*FlgD_26695 was checked by SDS-PAGE (Fig. 3a). The bands obtained from the crystallized sample and full length *Hp*FlgD_26695 were isolated and in gel digested with trypsin. The fractions of the extracted peptides were dried out, dissolved in 50% acetonitrile, supplemented with 0.1% formic acid and directly injected in the nano-ESI source. Mass measurements were performed with a quadrupole-TOF spectrometer (Waters, Manchester, UK) (capillary voltage: 2800–3000 V; cone voltage: 45 V; scan time: 1 s; interscan: 0.1 s). Analysis of the spectra was performed by using the MASSLYNX software (Micromass, Wynthenshow, UK). The data obtained from the mass analysis are presented in Fig. 4.

**Fig. 5:**
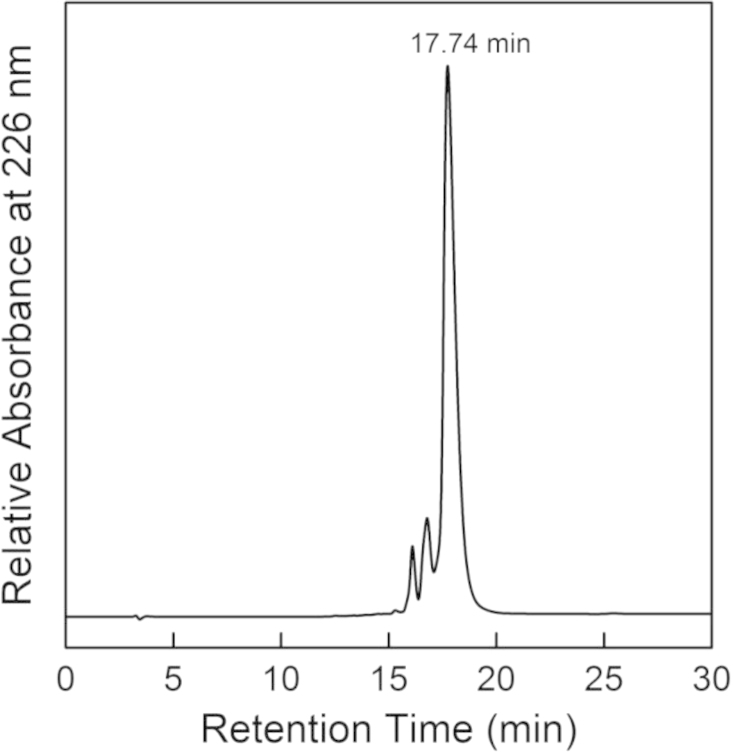
HPLC chromatogram of the full length *Hp*FlgD_G27. The major specie present in the solution corresponds to the size of 36,178 Da.

The mass of the *Hp*FlgD_G27 monomer was determined by mass analysis of the peaks isolated by reverse phase chromatography (C4-column, RP-HPLC), Fig. 5.

Presence of the His tag at the C-terminus of the full length *Hp*FlgD_26695 and crystallized *Hp*FlgD_26695 was evaluated with anti-His antibodies (Mouse monoclonal, 1:1000 dilution) and secondary antibodies (Goat anti-mouse HRP, 1:10,000) (Western blotting technique), Fig. 3b.

**Fig. 6:**
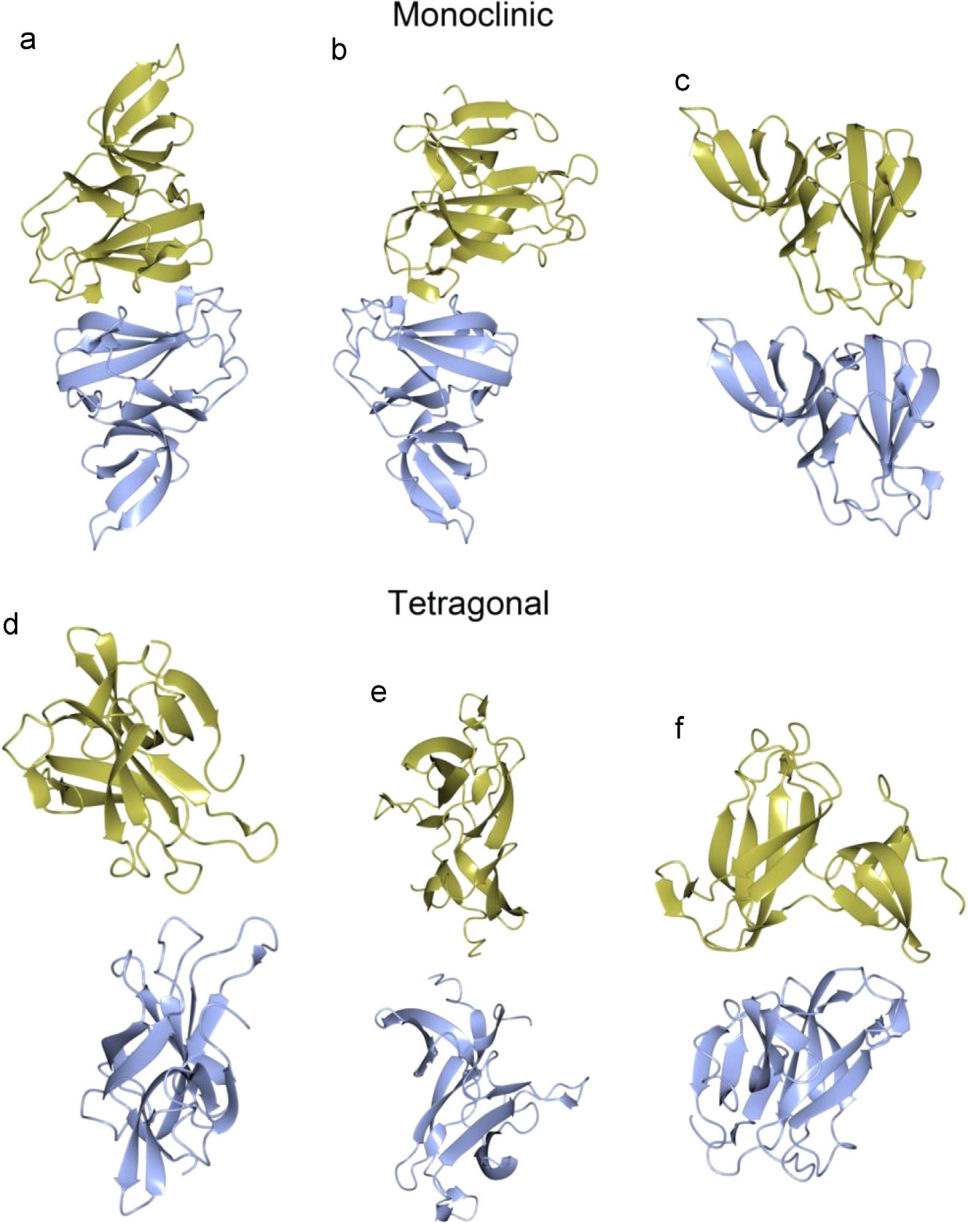
Different types of interfaces found between the molecules in the monoclinic crystal structure of *Hp*FlgD (a–c) and in the tetragonal crystal structure of *Hp*FlgD (d–f).

Fig. 6 shows different types of interfaces present in both crystal forms of *Hp*FlgD. In Table 2 the interface area, the number of hydrogen bonds and salt bridges involved in each interface are shown. The list of hydrogen bonds responsible for the tetramerization is presented in Table 3.

**Table 2:** Different types of interfaces between the molecules in the crystal structure of *Hp*FlgD. *N*_HB_ and *N*_*SB*_ refer to the number of hydrogen bonds and salt bridges, respectively. The interfaces labeled a−f with a* are shown in Fig. 6, while the interface labeled as t* refers to the interface responsible for the tetramerization (as shown in Figs. 4b and 6[1]).

Crystal system	Interface type	Monomer1···Monomer2[Symmetry code]	Interface area /Å^2^	*N*_HB_	*N*_SB_
**Monoclinic**					
	t*	B···A[x, y, z]	521.9	7	3
	t	D···C[−x, y, −z]	492.5	6	2
	t	B···A[−x, y, −z]	492.1	7	2
	t	C···D[x−1, y, z−1]	494.8	8	3
	a*	C···B[x, y, z]	302.3	–	–
	b*	D···B[−x+1, y, −z]	297.3	1	–
	c*	D···D[x, y−1, z]	280.1	1	5
	c	A···A[x, y−1, z]	268.2	1	5
	c	B···B[x, y−1, z]	216.4	3	5
	c	C···C[x, y−1, z]	194.3	3	5

**Tetragonal**					
	t	A···A[−y+1, x, z]	478.8	12	2
	d*	A···A[x, −y+1, −z]	299.9	4	4
	e*	A···A[−x,−y+1, z]	201.5	2	–
	f*	A···A[y−1/2, x+1/2, −z+1/2]	119.2	4	4

**Table 3:** Hydrogen bonds (Å) between monomers that are relevant for their assembly into a tetramer.

		***Hp*****FlgD_t**	***Hp*****FlgD_m**			
Monomer1	Monomer2[Symmetry code]	A···A[y+1, x, z]	B···A[−x, y, −z]	A···B	D···C[−x, y, −z]	C···D[x−1, y, z−1]
Ile264 [O]	Phe244 [N]	2.71	2.92	2.75	3.07	2.83
Phe266 [N]	Val242 [O]	2.88	2.82	2.80	2.94	2.90
Phe266 [O]	Val242 [N]	3.01	2.87	2.92	2.89	3.03
Glu265 [OE2]	Ser241 [OG]	2.93	3.36	3.20	3.15	3.17
*Glu265 [OE2]	Arg 252[ NH2]	3.43	2.79	2.61	2.72	2.85

* Denotes the salt bridge.

**Fig. 7:**
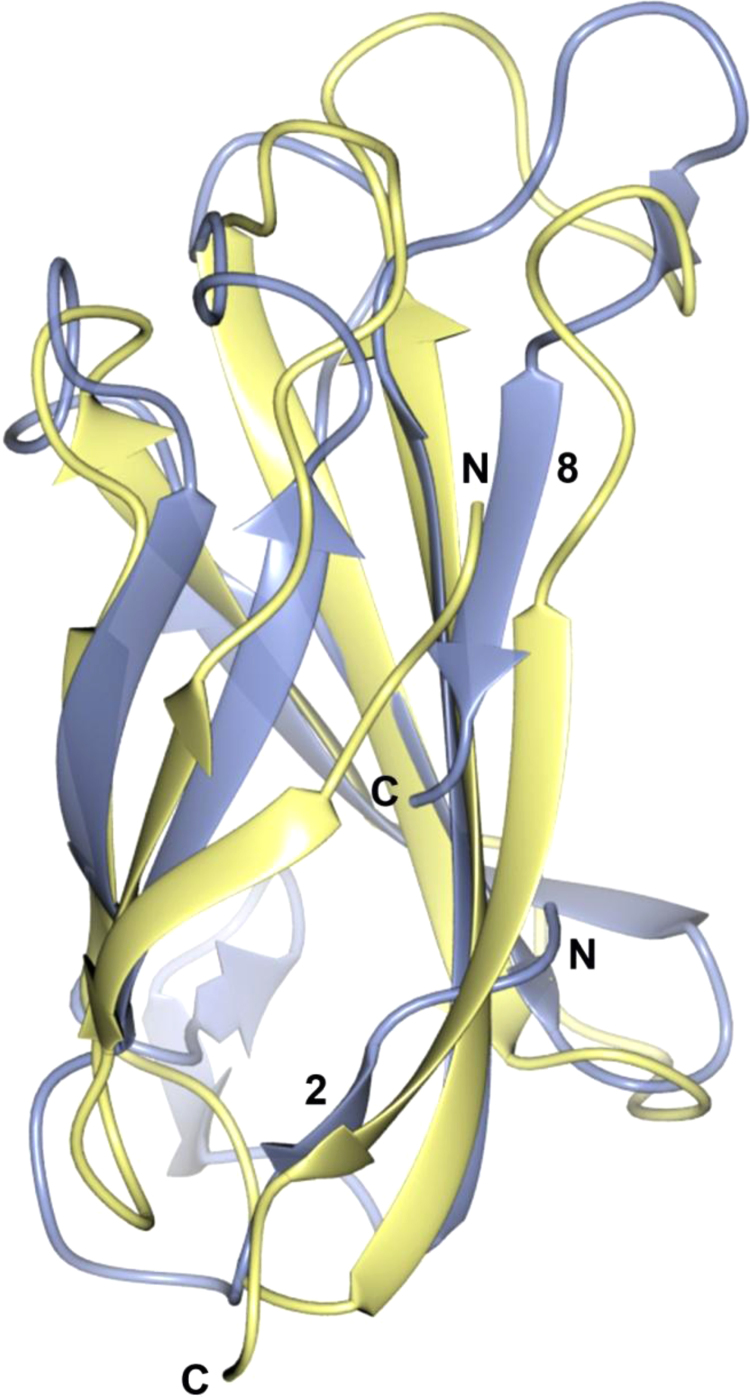
Superposition of the Fn-III domain in fibronectin (yellow) (PDB entry ID 1FNA) to the same domain in *Hp*FlgD_t (light blue). The r.m.s.d. for the superposition of 61 aligned C^α^ atoms of fibronectin on *Hp*FlgD_t is 2.51 Å.

**Fig. 8:**
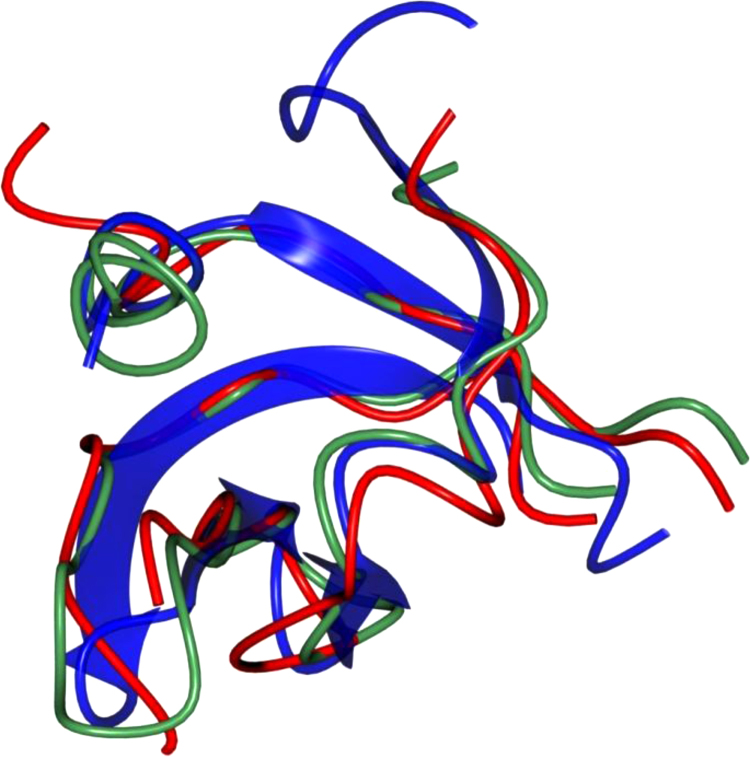
Superposition of the tudor domain in *Hp*FlgD_t (dark blue), *Xc*FlgD (green) and *Pa*FlgD_A (red). The r.m.s.d.s for the superposition of 47 aligned C^α^ atoms of *Xc*FlgD on *Hp*FlgD_t and 43 aligned C^α^ atoms of *Pa*FlgD_A on *Hp*FlgD_t are 2.09 Å and 1.55 Å, respectively.

Superposition of the Fn-III domain in *Hp*FlgD with the fibronectin domain in 1FNA [3] is presented in Fig. 7, while the superposition of the tudor domain in *Hp*FlgD and the same domain in *Pa*FlgD (PDB ID: 3OSV, [4]) and *Xc*FlgD (PDB ID: 3C12, [5]) can be seen in Fig. 8.

**Fig. 9:**
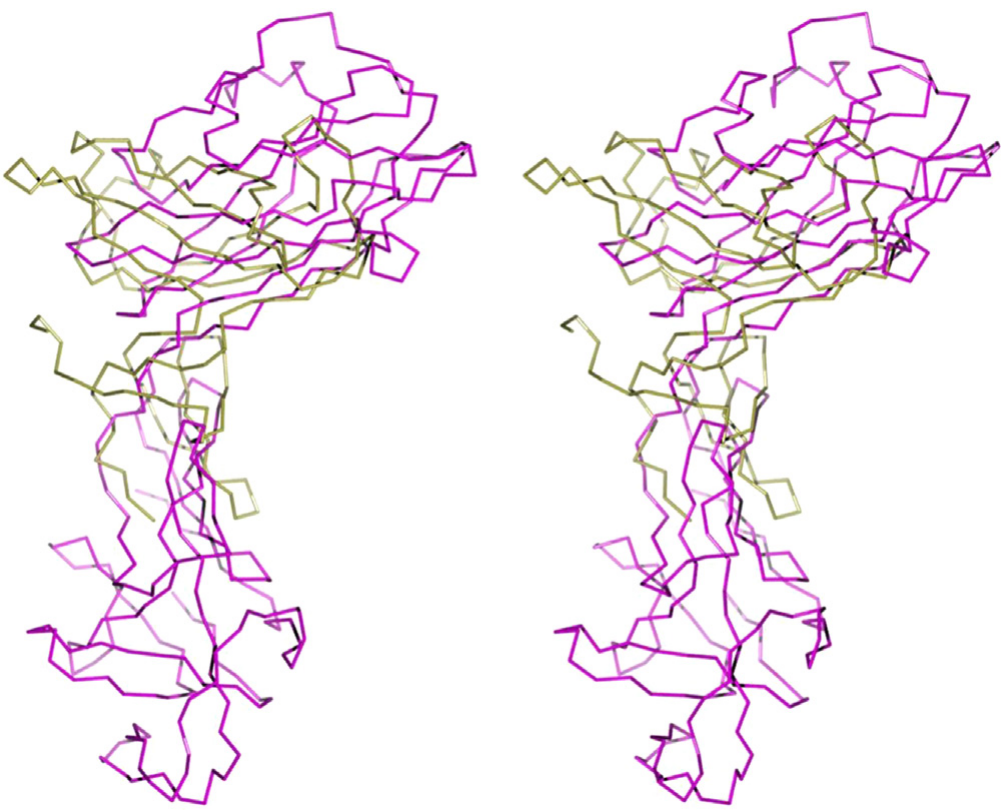
Stereoview of the superposed C^α^ chain trace of *Hp*FlgD_t (gold) and the modeled *Hp*FlgE (purple). The r.m.s.d. for the superposition of 68 aligned C^α^ atoms of modeled *Hp*FlgE on *Hp*FlgD_t is 3.44 Å.

Fig. 9 presents the overlayed structures of *Hp*FlgD and modeled *Hp*FlgE. Modeled *Hp*FlgE was prepared by homology using software Phyre^2^ [6].
